# Spontaneous Excitation Patterns Computed for Axons with Injury-like Impairments of Sodium Channels and Na/K Pumps

**DOI:** 10.1371/journal.pcbi.1002664

**Published:** 2012-09-13

**Authors:** Na Yu, Catherine E. Morris, Béla Joós, André Longtin

**Affiliations:** 1 Department of Physics, University of Ottawa, Ottawa, Ontario, Canada; 2 Ottawa Hospital Research Institute, Ottawa, Ontario, Canada; George Mason University, School of Computational Sciences and The Krasnow Institute for Advanced Studies, United States of America

## Abstract

In injured neurons, “leaky” voltage-gated sodium channels (Nav) underlie dysfunctional excitability that ranges from spontaneous subthreshold oscillations (STO), to ectopic (sometimes paroxysmal) excitation, to depolarizing block. In recombinant systems, mechanical injury to Nav1.6-rich membranes causes cytoplasmic Na^+^-loading and “Nav-CLS”, i.e., coupled left-(hyperpolarizing)-shift of Nav activation and availability. Metabolic injury of hippocampal neurons (epileptic discharge) results in comparable impairment: left-shifted activation and availability and hence left-shifted *I_Na-window_*. A recent computation study revealed that CLS-based *I_Na-window_* left-shift dissipates ion gradients and impairs excitability. Here, via dynamical analyses, we focus on sustained excitability patterns in mildly damaged nodes, in particular with more realistic Gaussian-distributed Nav-CLS to mimic “smeared” injury intensity. Since our interest is axons that might survive injury, pumps (sine qua non for live axons) are included. In some simulations, pump efficacy and system volumes are varied. Impacts of current noise inputs are also characterized. The diverse modes of spontaneous rhythmic activity evident in these scenarios are studied using bifurcation analysis. For “mild CLS injury”, a prominent feature is slow pump/leak-mediated *E_Ion_* oscillations. These slow oscillations yield dynamic firing thresholds that underlie complex voltage STO and bursting behaviors. Thus, Nav-CLS, a biophysically justified mode of injury, in parallel with functioning pumps, robustly engenders an emergent slow process that triggers a plethora of pathological excitability patterns. This minimalist “device” could have physiological analogs. At first nodes of Ranvier and at nociceptors, e.g., localized lipid-tuning that modulated Nav midpoints could produce Nav-CLS, as could co-expression of appropriately differing Nav isoforms.

## Author Summary

Nerve cells damaged by trauma, stroke, epilepsy, inflammatory conditions etc, have chronically leaky sodium channels that eventually kill. The usual job of sodium channels is to make brief voltage signals –action potentials– for long distance propagation. After sodium channels open to generate action potentials, sodium pumps work harder to re-establish the intracellular/extracellular sodium imbalance that is, literally, the neuron's battery for firing action potentials. Wherever tissue damage renders membranes overly fluid, we hypothesize, sodium channels become chronically leaky. Our experimental findings justify this. In fluidized membranes, sodium channel voltage sensors respond too easily, letting channels spend too much time open. Channels leak, pumps respond. By mathematical modeling, we show that in damaged channel-rich membranes the continual pump/leak counterplay would trigger the kinds of bizarre intermittent action potential bursts typical of injured neurons. Arising ectopically from injury regions, such neuropathic firing is unrelated to events in the external world. Drugs that can silence these deleterious electrical barrages without blocking healthy action potentials are needed. If fluidized membranes house the problematic leaky sodium channels, then drug side effects could be diminished by using drugs that accumulate most avidly into fluidized membranes, and that bind their targets with highest affinity there.

## Introduction

**Figure 1:**
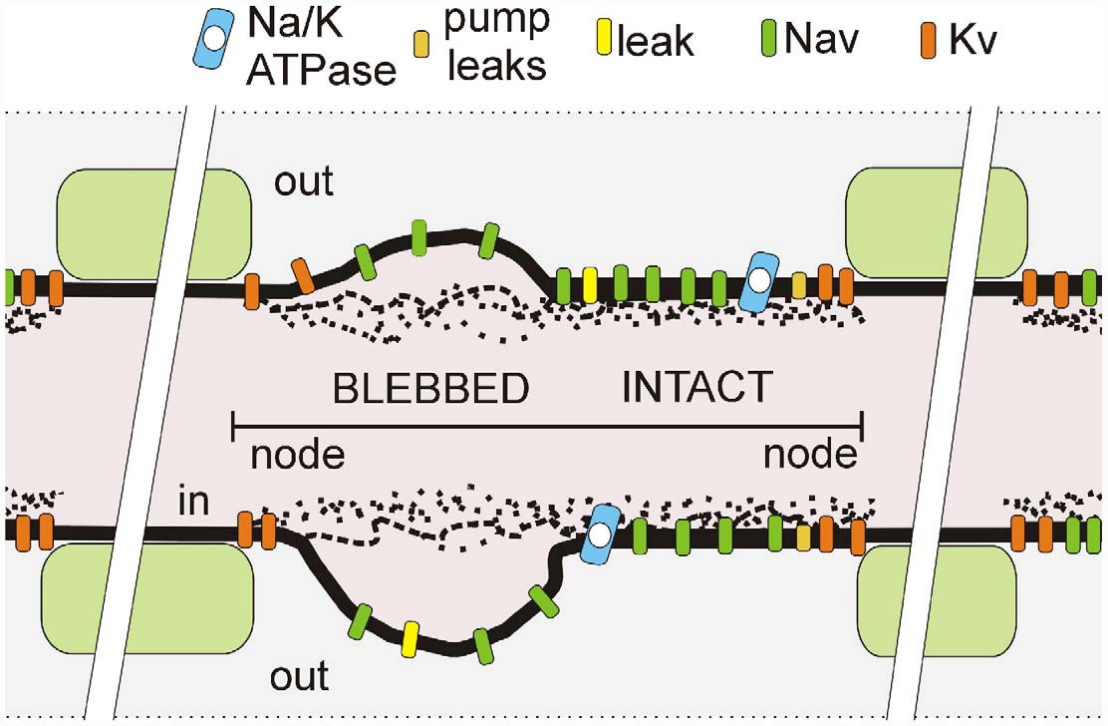
Schematic of a mechanically-injured node of Ranvier depicted with a mix of intact-looking and severely-blebbed axolemma (as labelled) such as seen in transmission electromicrographs of stretch-injured optic nerve nodes [2]. In pipette aspiration bleb injury, the cortical actomyosin-spectrin skeleton progressively detaches [6]. Our model considers a node as one equipotential compartment in which actual spatial arrangements of pumps and channels are irrelevant. However, the fraction of Nav channels in the injured portion of the membrane, along with the severity of their gating abnormality, are model parameters.

In any healthy sodium channel (Nav)-rich plasma-membrane, the bilayer is a far-from-equilibrium nanostructure that degrades wherever mechanical or chemical insult causes the inner leaflet to detach from adherent cortical cytoskeleton [1]–[4]. Severe insults cause readily-visualized rounded blebs of disordered, fluidized bilayer (see Figure 1), while milder damage causes intermediate degrees of disordered “bleb-like” injury [2], [5], [6]. Though many membrane proteins would be affected, Nav channels are overwhelmingly the key players in many excitable membranes, including nodes of Ranvier [1]. Positive-feedback Nav currents yield action potentials (APs) that dissipate Na/K gradients maintained by Na/K-ATPases so not surprisingly, membrane-damaging conditions (e.g., trauma, ischemia, muscular dystrophy) that render Nav channels chronically leaky trigger excitotoxic cellular demise [3], [7].

Pipette aspiration electrophysiology studies on Nav1.6-rich membranes (patch-clamped *Xenopus* oocyte patches) [8] showed that the aspiration-induced bleb-like injury causes “left-shift Nav-leak”; progressive aspiration damage irreversibly and progressively shifts the voltage midpoint of *g_Na_(V)* in the hyperpolarizing direction. When maximal disorder is reached, the irreversible shifting process “saturates” and any further aspiration-induced left-shifts are reversible (see also [9], [10]). Nav-availability depends on fast inactivation, a transition limited by fast activation [10]. Accordingly, damage causes equal-magnitude left-shifts for *g_Na_(V)* and availability(*V*), thereby left-shifting the window conductance. The shifted steady-state (window) current constitutes a Nav-leak or abnormal “subthreshold persistent current”, the entity we describe as coupled left-shift (CLS) Nav-leak [11]. Pipette aspiration also causes irreversible Nav-CLS for Nav1.4 and Nav1.7 [12], [13] (preparation: oocytes) and Nav1.5 channels [14] (preparation: HEK cells).

In hippocampal neurons, a Nav-CLS based window current left-shift occurs following metabolic insult from prolonged epilepsy-like stimulation [15]. Hippocampal neurons exposed to the industrial compound, melamine, also show Nav-CLS and exhibit hyperexcitability [16]. In neurons and muscle fibers, left-shift of Nav availability (i.e., steady-state inactivation) [17] is reported for many damaging and/or toxic conditions (see Table 1 of [3]) and for membrane fluidizing conditions [18]; fast activation also becomes left-shifted in muscles (e.g. [19]) and neurons (e.g. [20]). As predicted when membrane injury causes Nav-CLS [11], sick excitable cell pathologies range from hyperexcitability through ectopic excitation to depolarizing block. Sucrose-gap voltage clamp of nodes of Ranvier subjected to ischemic and other insults could, we have suggested [3], be used to test to what extent the initial Nav-leak of injured Nav-rich native membranes is explained by Nav-CLS. Injured-bilayer *I_CLS-window_* could help explain [3] why, invariably, clinical Nav inhibitors protective against excitotoxicity are lipophilic [21]; moreover, if bilayer structure matters, then retuning therapeutic strategies to better target channels in damaged bilayer could pay dividends [3].

**Table 1:** Parameters for node of Ranvier with pump.

Membrane capacitance	*C* = 1 µF/cm^2^
Maximal *Na^+^*conductance	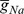 = 120 mS/cm^2^
Maximal *K^+^* conductance	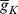 = 36 mS/cm^2^
Faraday constant	F = 96485.3399
Temperature	*T* = 293.15 K
Volume of inside compartment	*Vol_i_* = 10^−15^ m^3^
Volume of outside compartment	*Vol_o_* = 10^−15^ m^3^
Surface area	A = 6×10^−8^ cm^2^
*K^+^* pump leak conductance	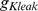 = 0.1 mS/cm^2^
*Na^+^* pump leak conductance	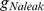 = 0.2 mS/cm^2^
Leak conductance	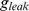 = 0.5 mS/cm^2^
Leak reversal potential	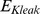 = −59.9 mV
Maximum pump current range used	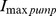 = 15 to 156 µA/cm^2^
(standard value, except as noted)	= 95 µA/cm^2^
Pump *K^+^*-dissociation constant	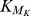 = 3.5 mM
Pump *Na^+^*-dissociation constant	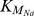 = 10 mM

Here, our goal is to understand in dynamical terms the mechanisms of abnormal steady-state excitability in membrane modeled with CLS-type injuries of various intensities. In particular, we wish to determine if – in conjunction with axolemmal Na/K pumps – CLS-injury predicts forms of abnormal excitability reported for “mildly” injured excitable cells, i.e., cells that, with appropriate remediation, could be salvaged. In healthy unstimulated axons, quiescence is the norm and spontaneous firing is neuropathic and ectopic.

Several recent studies involving simulation and dynamical modeling addressed abnormal or paroxysmal discharge in injured neurons. Injured DRG neuron simulations [22] showed that subthreshold oscillations (STOs) resembling ones recorded in vivo and relevant to neuropathic pain can be generated via depolarizing stimuli to a membrane with three distinctive *g_Na_*s. Importantly, though, for STOs, the slower two *g_Na_*s required first order, rather than the usual third order activation kinetics. We address this later. Similarly, for two Nav-type and two Kv-type conductances, Choi and Waxman [23] showed responses to current injection (mimicking sensory input) that included STOs with the spikes; there too, for STOs to occur, one of the *g_Na_*s required first-order activation kinetics.

STOs typically occur in conjunction with bursts of spikes, appearing in healthy neurons, (e.g., entorhinal cortex [24]) as small *V_m_* oscillations for a discrete range of conditions. Dynamically-speaking, STOs emerge where there is bistability between tonic firing and quiescence [25]. For allodynia-related neuropathic pain, a dynamical study of firing patterns showed *E_Anion_* changes having a key stability role [26]. Firing abnormalities of demyelinated axon were mimicked in a dynamical modeling study with fixed Nernst potentials and *g_Na_/g_leak_* ratios varied [27]. Regarding paroxysmal spiking, the same group [28] explored initiation and termination of afterdischarges and spontaneous activity; there, a non-inactivating *g_Na_* induced bistability and to mimic [ion] homeostasis following transient Na^+^-loading, an exponential decay provided a slow relaxation toward initial [*Na* ^+^]_intracellular_. Käger *et al* [29] investigated epilepsy-like spontaneous bursting patterns and elevated [*K* ^+^]_extracellular_, and Barreto, Cressman and colleagues [30], [31] performed a dynamical study for oscillating [ion]s connected with epileptic seizures.

Our Nav-CLS-based model of membrane injury uses a Hodgkin-Huxley axon without or with a Na/K pump as developed previously in [11]. That model focused on transitory responses to abrupt CLS “injury” (left-shift intensity and the fraction of total *g_Na_* affected were varied) and revealed how CLS-injury affects *V_m_(t)* and ion homeostasis. Once the injured system (no pumps) stabilized, depolarizing current was applied, and based on spontaneous and stimulated behaviors, Nav-CLS injury activity was classified under various regimes (Figures 5 and 9 in [11]). CLS-injury caused hyperexcitability, ectopic excitation or depolarizing block, and interfered with saltatory propagation of normal action potential (AP) traffic. Varying extracellular volume can significantly alter the quantitative measures of the bursting, consistent with a major role for pump activity. Unexpectedly, it was discovered that Nav-CLS in conjunction with pumps could generate STOs. Since injured neuron neuropathic pain discharge is characterized by diverse STO phenomena, we undertook the dynamical analysis of Nav-CLS injury presented here.

We model acute injury: even in egregiously blebbed native membranes, Nav channels retain their ability to respond to voltage [32], [33]. Keeping *g_Na-max_* constant corresponds to an absence of *de novo* Nav channel expression or membrane remodelling. Immunochemistry confirms an abundance of axolemmal Nav channels at damaged nerve-end neuromas, sites considered to be loci of ectopic excitation [34]. Our CLS-injury model is consistent with present views of voltage-gated channel structure/function [35]–[37]. Nav-CLS injury *in situ* would likely be of non-uniform intensity, a point we address computationally in several ways. Except for some instances of modified pump intensity, “impairment” refers exclusively to Nav-CLS.

Na/K pump activity is sensitive to ATP levels, injury and bilayer lipid characteristics [38]–[40], but how mild membrane injury would affect pump rates is unclear. So here we vary the maximal pump rate to explore its effect on tonic firing and bursting (in this paper, both firing patterns are spontaneous).

The diverse spontaneous steady-state rhythmic activity patterns that became evident in our injury scenarios are studied using bifurcation analyses. For mild CLS injury, a prominent and robust feature was the emergent slow process of pump/leak-mediated *E_Ion_* oscillations [11]. As we will illustrate, these slowly oscillating *E_Ion_* values cause time-dependent changes in the firing thresholds, which in turn will lead to complex spontaneous STO and bursting behaviors; the complexity of the observed patterns is further exacerbated by current noise.

In summary, we study, via dynamical analysis, a simple, robust, and biophysically explicable model of mild axonal injury in which pumps remain functional, i.e., we examine a collection of mild Nav-CLS injury scenarios. The major finding is that interactions between the window current based Nav-leak and Na/K pump activity engender slow oscillations of the Na^+^ and K^+^ driving forces that spontaneously trigger a plethora of known neuropathological excitability patterns. The following results are based on simulations of a Nav-CLS model of injured node described in the *Methods* section and in Table 1 which is a list of parameters.

## Results

### Membrane potential oscillations in CLS injured nodes for fixed *E_ion_*

Simulated nodes with Nav-CLS injury were shown [11] to exhibit activity regimes that include tonic and burst firing, plus a quiescent steady state. In this section we explore the node with simulated more nuanced versions of Nav-CLS injury and find that rhythmic firing combined with small-amplitude *V_m_* oscillations are commonplace. We start with cases of constant [*Ion*] (pump turned off), approximating axonal situations over brief times. We choose cases that provide snapshots of dynamics at selected fixed reversal potentials that could occur at different stages of injury. For fixed reversal potentials (*E_Ion_*) we take three sets of values corresponding to different transmembrane *Na^+^* and *K^+^* gradients: (i) *E_Na_* = 50 mV, *E_K_* = −77 mV; (ii) *E_Na_* = 42 mV, *E_K_* = −77 mV when initial injury has elicited a change in *I_Na_* thence [*Na* ^+^]s; and (iii) *E_Na_* = 42 mV, *E_K_* = −71 mV. In this latter case, *I_Kpump_*, because of its dependence on [*Na* ^+^]s, changes, which results in *E_K_* changes.

**Figure 2:**
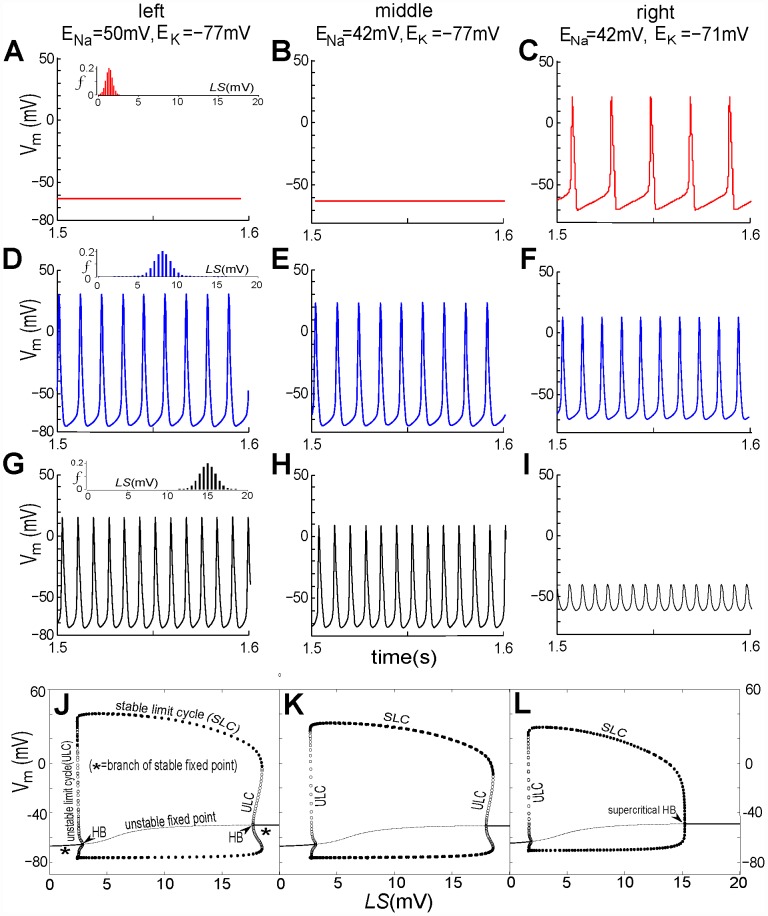
Nav-CLS induced spontaneous activities of injured node: steady state with fixed *E_Ion_*, tonic spiking, tonic subthreshold oscillations (STOs). By setting *I_maxpump_*, *g_Naleak_*, and *g_Kleak_* to zero and eliminating Eqs (11) and (12), the *E_Ion_* are artificially maintained at fixed values. Three sets of values are considered: left column, *E_Na_* = 50 mV, *E_K_* = −77 mV; middle column: *E_Na_* = 42 mV, *E_K_* = −77 mV; right column: *E_Na_* = 42 mV, *E_K_* = −71 mV. For the first 3 rows, Nav-CLS have Gaussian distributions (mean±SD): (**A,B,C**) 1.3±0.4 mV; (**D,E,F**) 8±1 mV; (**G,H,I**) 15±1 mV. For the last rows,(**J,K,L**) bifurcation diagrams (solution of *V_m_* in terms of *LS*) are plotted, and there, for computational tractability, single *g_Na_* populations (i.e. *f* = 1, no Gaussian “smears”) are used. As labeled, the solid line, dashed line, filled dots, open dots and “HB” respectively denote: stable fixed point (i.e. resting potential (RP)), unstable fixed point, stable limit cycle (SLC, i.e., tonically firing APs), unstable limit cycle (ULC) and Hopf bifurcation point (HB). When *E_Na_* alone changes (**J** to **K**), the bifurcation structure shows only slight changes in the amplitude of SLC and the locations of subcritical HB on both ends. When *E_K_* changes (**K** to **L**), the HBs on both ends shift to the left (i.e. towards relatively smaller *E_Na_*) and the previously subcritical HB (right side) becomes supercritical. Across the 3 columns, note that if the system did have pumps, interactions between the *E_Ion_* and *I_pump_* would continually and slowly change *E_Ion_* thereby repeatedly evoking these activity patterns.

Nav-CLS intensities induced by membrane damage would vary within and between axons, as suggested in Figure 8 Aiii in [8]. To reflect this, three sets of *LS_i_* and *f_i_* are applied. To incorporate the idea of “smeared-out LS”, the fraction *f_i_* of the population are given a Gaussian distribution of LS with mean±SD of 1.3±0.4 mV, 8±1 mV and 15±1 mV. The numerical results are plotted in Figure 2. For the first two *E_Ion_* pairs (i) and (ii) (left and middle columns), *V_i_* dynamics in the injured node vary from quiescence to repetitive spiking, with the firing rate increasing and the spike amplitude decreasing as mean(*LS*) increases. However, for *E_Ion_* pair (iii) (right column), this node shows changes from STF at high amplitude (again, with increasing firing rate and decreasing amplitude) to a very low amplitude oscillation (Figure 2I) that shares features of subthreshold oscillations (STOs).

The question of what happens to the threshold for STF for these changes in *E_Ion_* is raised by these results. The bottom row, panels J-K-L, displays the bifurcation diagram of the voltage excursions as a function of left-shift; the *E_Ion_* values for each panel are those used in the three panels above it. For these diagrams, the left shift is the bifurcation parameter; all channels (*f* = 1) assume this shift (there is no spread, i.e. SD (standard deviation) = 0). Each diagram is constructed by choosing a value of *LS*, running a simulation, letting transient behavior die out, then measuring various characteristics of the steady-state voltage solution. In particular, here we measure the value of a fixed point (resting potential, RP) or the minimum and maximum values of a *V_m_* oscillation. This procedure is repeated over a range of *LS* values, and the characteristic values are plotted against LS.

As *LS* increases, we see in panels J, K, and L a Hopf bifurcation (HB) i.e. a transition between a stable resting potential (RP), or in the diagram, stable fixed point, and tonic firing. Further, there is a narrow range of *LS* where both RP and tonic firing coexist; this is known as a “subcritical” HB. Subcritical HBs are seen on the left part of each diagram, and on the right part of diagrams J and K. In panel L however, the right part displays no such coexistence, and the HB is “supercritical”. This means that as *LS* increases further, there is an abrupt (although continuous) transition from tonic firing to RP. Upon decreasing *E_Na_* (panels J to K), the threshold moves to higher *LS* values, and the spike height is reduced. However, when *E_K_* is reduced (panel L), the threshold moves to a lower *LS*, which explains the onset of firing in panel C. The decrease of the repolarizing current also continuously raises the minima and lowers the maxima of the voltage excursions. This underlies the trend to low amplitude spiking behavior (cf. panels C→F→I). Thus, in axons where injury has caused Nav-CLS and is fostering gradual rundown (depletion) of the ion gradients, a range of abnormal excitability patterns could be expected to appear then disappear.

Note that the mean *LS* in Figure 2I corresponds to the limit cycle regime (Figure 2L) – although it is very close to the fixed point (quiescent) regime which is to the right of the supercritical Hopf bifurcation in Figure 2L. This is why the spike amplitude is so small – but technically they are still spikes, not STOs; this would be the case even if the Hopf bifurcation were subcritical (as in the right part of Figures 2J and K). There is no injected current here, and the system can generate tonic firing of low amplitude spikes, so we cannot speak of depolarization block. However, depolarization block can be produced by injecting a current of sufficient magnitude (not shown).

Since the *E_Ion_* are controlled by intra- and extracellular [*Ion*]s (Eq (13)) which in turn depend on pump currents via Eq (10), these results imply: a) *I_pump_* will substantially affect excitability at injured nodes [41], and b) in the presence of a small rundown of the K^+^ gradient, Nav-CLS is sufficient to produce STO-like voltage excursions. We will see in the two sections on bursting below (*Bursting in injured nodes* and *Bifurcation analysis of bursting*) that these excursions and STOs share a common origin.

### *V_m_* oscillations in a CLS injured node with the pumps on

Whereas the transition from quiescence to tonic firing when *E_K_* moves closer to zero (Figure 2B to 2C) is expected, since the move causes a small depolarization, the appearance of STO-like excursions (Figure 2H to 2I) is less intuitively obvious. To better understand how pump current affects the emergence of small oscillations, we simplify the above multiple Gaussian-distributed *g_Na_* populations by forming two subpopulations: *LS_i_* = [0,15]mV and *f_i_* = [0.5,0.5], signifying that half of Nav channels have no shift, and the rest have a 15 mV CLS. This choice for *g_Na_* populations is taken from the tonic firing regime (Figure 9B in [11]) that occupies the largest part (∼40%) portion of the *LS/f* plane depicting five spontaneous activity regimes of injured nodes. We turn on the Na/K pump and mimic injury to pump function by varying the parameter *I_maxpump_* in Eq (10). *I_maxpump_* is a functional maximum, a rate that, as per Eq 13 would only be attained for [*K^+^*]_o_ = 0 and [*Na^+^*]_i_ = 0 (unrealistic in living systems). It differs among axons [42] and will vary with the cellular surface area, Na/K-ATPase density, bilayer lipid composition [39], [42] and fluidity [43], and temperature [44] among other factor [45]. Importantly, *I_maxpump_* can be reversibly decreased experimentally by specific inhibitors (e.g. strophanthidin, ouabain).

**Figure 3:**
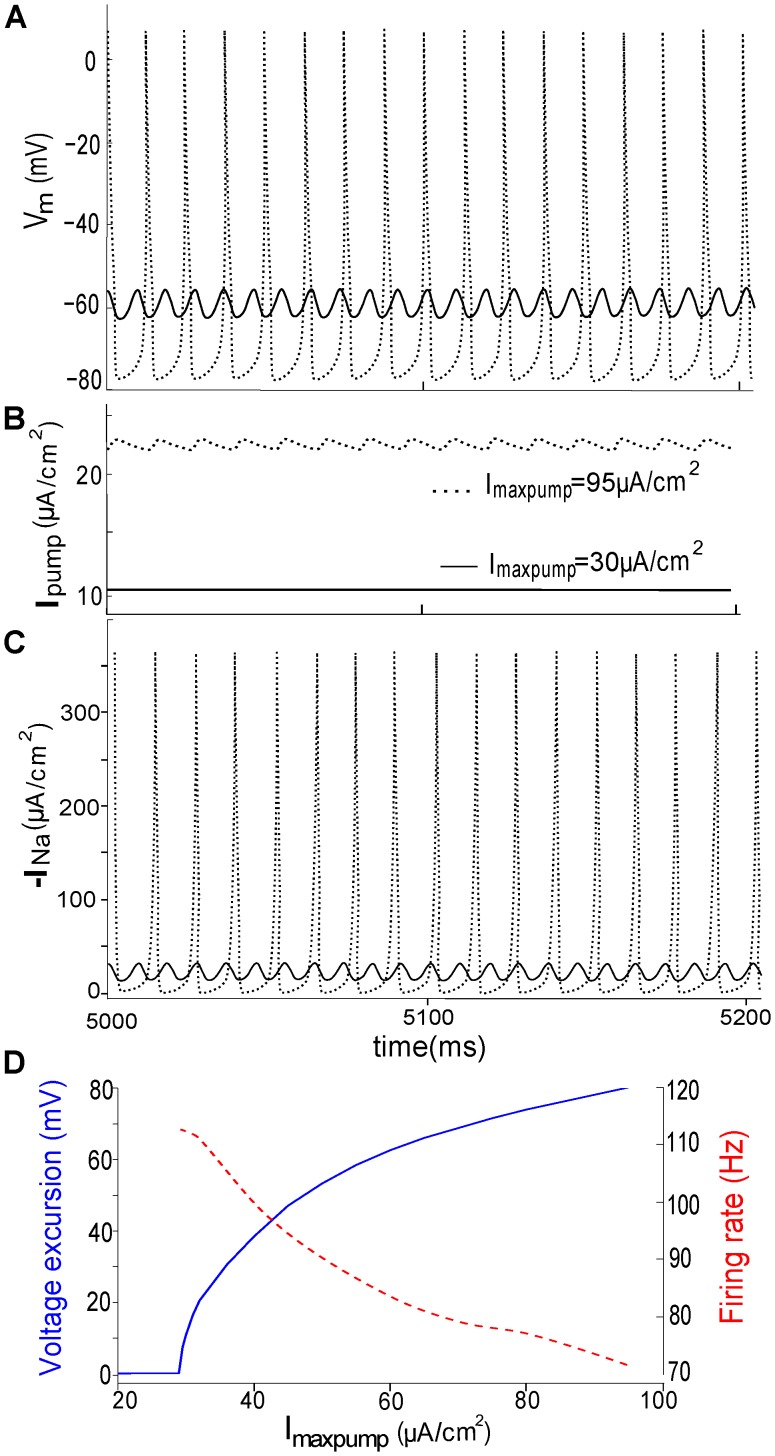
Ectopic steady-state *V_m_* excursions are tuned by *I_pump_*. (**A**) From the spontaneous tonic firing regime of Figure 9 in [11], a random point (*LS_i_* = [0,15]mV, *f_i_* = [0.5,0.5]) is selected; as illustrated, *V_m_* oscillation amplitude varies as *I_maxpump_* is set at 30 and 95 µA/cm^2^. (**B**) *I_pump_* changes from a constant 10 µA/cm^2^ (associated with a small periodic *V_m_* fluctuation) to a fluctuating 23±0.5 µA/cm^2^ (associated with a train of APs). (**C**) Corresponding total *I_Na_*. (**D**) With growing *I_maxpump_*, voltage excursions (blue) increase and their oscillation frequencies (red) decrease.

Figure 3 demonstrates that when *I_maxpump_* falls from 95 to 30 µA/cm^2^, repetitive AP activity (dotted line) is replaced by tonic low-amplitude *V_m_* oscillations (solid line). While the *V_m_* -oscillation amplitude increases as *I_maxpump_* increases (Figure 3D), the firing rate decreases. This suggests that, if *I_pump_* were to decrease, repetitive APs could change to STOs. Inhibiting the Na/K pump can further lead to a complete cessation of firing. This is in general agreement with experimental results [42]. It is noteworthy that because [*Na^+^*]_i_ and [*K^+^*]_o_ (Eq (10)) limit maximal pump current, the upper values of *I_pump_* are considerably less than *I_maxpump_*. In Figure 3B, e.g., with *I_maxpump_* at 95 µA/cm^2^, *I_pump_* fluctuates around 22.7±0.5 µA/cm^2^. The section *Effects of varying maximal pump current* discusses the impact of *I_maxpump_* on the bursting regime.

### Bursting in injured nodes

After repetitive firing activity, the electrogenic Na/K pump mediates the restoration of the ion gradients on a relatively slower time scale. Figures 2E and 2F suggest that pump current, by driving *E_Ion_* changes, could also induce patterns of bursting in which volleys of high-amplitude spikes are separated by periods of low-amplitude STOs.

**Figure 4:**
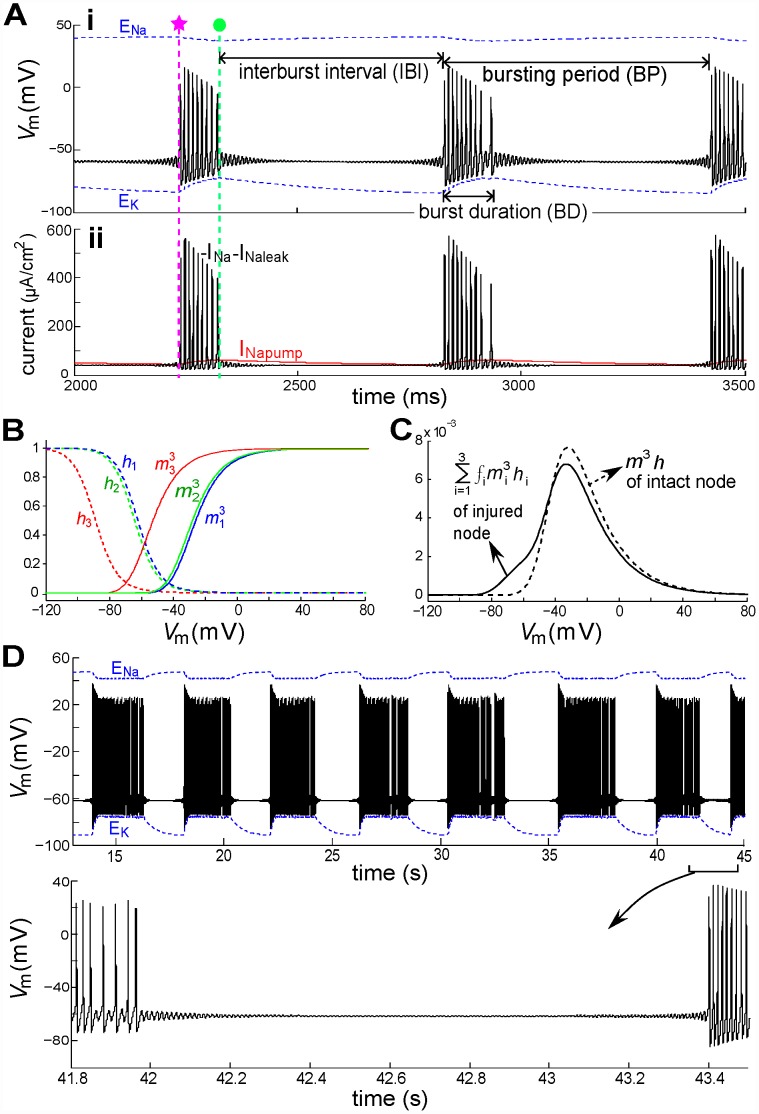
Transitions between STO and burst behaviors. (**A**) Upper: *V_m_* (black solid line) at an injured node and the varying *E_Ion_* (*E_Na_* and *E_K_*; blue dotted lines). Three *g_Na_* populations are used: *LS_i_* = [0,2,26.5]mV and *f_i_* = [0.72,0.08,0.2] with *Vol_i_* = *Vol_o_* = 10^−15^ m^3^. Initiation and termination times of a burst of spikes (pink star, green dot, respectively) are used in Figure 5. Lower: corresponding Na^+^ currents, as labelled. (**B**) Equilibrium values of Nav channel activation and inactivation variables, *m_i_* and *h_i_*. (**C**) The steady-state open probabilities, 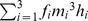: with a mild injury *g_window_*(V) magnitude is slightly less at 0 mV (vertical line and circles) but much enlarged at voltages near the normal RP (−65.5 mV for fixed [ion] condition). (**D**) Another example: *LS_i_* = [0,2,20]mV and *f_i_* = [0.72,0.08,0.2] and *Vol_i_* = *Vol_o_* = 3×10^−15^ m^3^, with expanded detail showing STOs. Note the difference of time scales in (A) and (D), reflecting the fact that a 3-fold lower axonal surface-to-volume ratio in (D) slows the rate of [ion] changes.

In this section we pursue this idea by simulating nodes with three *g_Na_* populations (*LS_i_* = [26.5,2,0]mV and *f_i_* = [0.2,0.08,0.72]) and *I_maxpump_* set at 95 µA/cm^2^. In Figure 4A a node injured this way produces bursts of spikes separated by STOs. The silent and active phases of such bursts are associated with pump current dynamics and the left-shifted window conductance (*m^3^ h*). In the silent phase where *V_m_* is near the RP (−60 mV), the Nav-CLS channels are a “leaky” Nav component (Figure 4B and 4C) flowing into the injured node, but without depolarizing it above firing threshold, because outward *I_Napump_* slightly exceeds the inward sodium current (the sum of *I_Na_* and *I_Naleak_*). The result: *V_m_* is destabilized and STOs develop. *I_Napump_* keeps decreasing (Figure 4Aii), becoming less than inward sodium current at about the time marked (pink star) in Figure 4Ai. Therefore *Na^+^* accumulates inside and *K^+^* outside the node, resulting in *E_Na_* and *E_K_* changes, based on Eq (13).

When changes in *E_Ion_* further reduce the firing threshold (system dynamics will be explained in the following section) the injured node would cross the diminished threshold and produce APs. With the consequent [*Na^+^*]_i_ increase, *I_Napump_* starts increasing. It eventually prevents the node from crossing the threshold and APs cease (green dot in Figure 4Aii) and *V_m_* falls back to the STO state around the RP. Repeated switching between clusters of spikes and STO intervals constitutes a form of bursting behavior that, given the involvement of the pump, should be sensitive to the surface to volume ratio, as per the numerical results of Käger *et al* [29]. With intracellular and extracellular nodal volumes tripled (*Vol_i_* = *Vol_o_* = 3×10^−9^ m^3^) and the surface area fixed (6×10^−8^ m^3^) (Figure 4D), bursting period (BP – from the beginning of one burst to the beginning of the next) and burst duration (BD) (Figure 4Ai) are prolonged. This confirms that, for the Nav-CLS model of injury, both the burst parameters are indeed sensitive to the surface-to-volume ratio.

**Figure 5:**
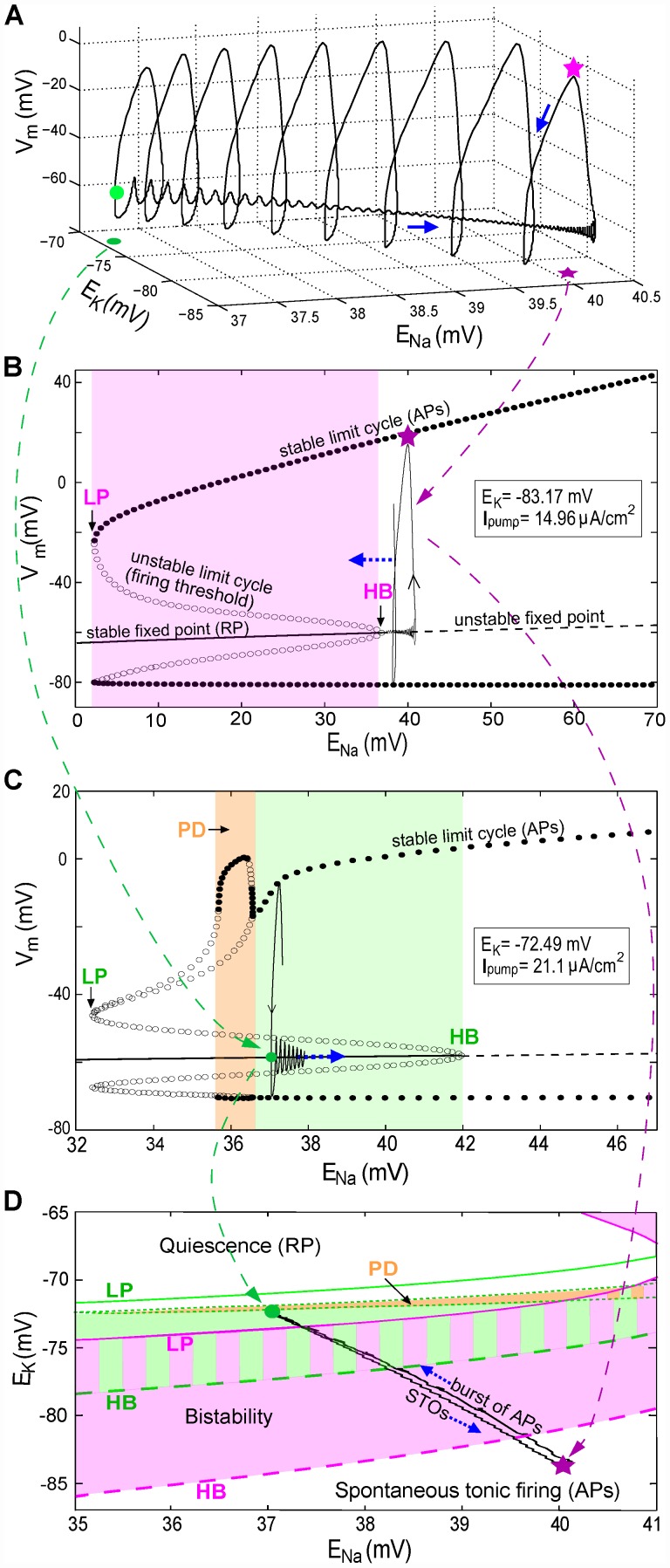
Burst dynamics explained with three-dimensional *V_m_* trajectories and bifurcation diagrams (for bursts in Figure 4A with *I_maxpump_* = 95 µA/cm^2^). Note: x-axis scales are different is each plot. (**A**) The first burst of spikes in Figure 4A plotted as a function of time are now plotted in 3-D as a function of *E_Na_* and *E_K_* (blue arrow: direction of *V_m_* trajectory). (**B**) Bifurcation diagram for fixed *E_K_* and *I_pump_* as per Figure 4A pink star; *E_Na_* is the slowly varying parameter. Lines and points are labeled (see also abbreviation list) and have the same meanings in (C). For *E_Na_* at its Figure 4A pink star value (40.2 mV), the only stable solution is a periodic orbit (pink oval), with each cycle corresponding to a spike (in a burst). The oval-loop symbolizes one cycle of *V_m_* oscillation at fixed *E_Na_*. During a burst the *E_Na_* decline shifts orbits leftward into the bistability regime (pink area) where there exist two stable solutions: a limit cycle and a stable fixed point. (**C**) Bifurcation diagram for *E_K_* and *I_pump_* fixed as per Figure 4A green dot. For *E_Na_* at its Figure 4A green dot value (37.27 mV), the system (large green dot) is within the bistability regime (green area). *V_m_*, attracted by this fixed point, has STOs (drawn as the green loop) until *E_Na_* increases and superthreshold-oscillations (spikes) return. The PD region corresponds to period-doubling bifurcations. (**D**) Two-parameter phase diagram for *E_K_* and *E_Na_*. Pink solid and dashed curves represent LP (saddle-node bifurcation) and HB, respectively, when *I_pump_* is fixed as in B. The green solid, dashed, and dash-dotted curves represent LP, HB and PD, respectively, when *I_pump_* is fixed as in C. With varying *I_pump_* the bistability regime shifts from the pink to the green area (the zone with both colors is the overlap of these two areas). The gray area between two green dash-dotted curves is a zone with PD bifurcations. The black loop shows *E_K_* and *E_Na_* orbits during a burst.

Interestingly, the solutions in Figures 4A and 4D do not exactly repeat. The number of spikes during the active phase of the burst varies slightly among bursts. Additionally, missed spikes at the end of the burst phase relate to the existence of period-doubled solutions (PD) as we will see in Figure 5C. Even though below we characterize them by an approximate bursting “period”, BP, these solutions are likely chaotic.

### Bifurcation analysis of bursting

As seen above, bursting is followed by quiescence and STO's are observed at the onset and offset of bursting. We now expose the dynamical structures underlying these behaviors. During firing, the ion gradients are slowly depleted due to the window current in the injured node. The pumps cannot compensate for the loss, and *E_Na_* and *E_K_* move towards zero. During quiescence the pumps replenish the concentration gradients, and the absolute values of *E_Na_* and *E_K_* increase. Spontaneous rhythmic bursts in Figure 4A are shown three-dimensionally in Figure 5A with dynamic (i.e. time-varying) *E_Ion_*. A bifurcation analysis based on a decomposition of the full dynamics into slowly and rapidly changing variables (e.g. *E_Ion_* and *V_m_* respectively, Figure 5B and 5C) helps identify the burst mechanism. This is known as a slow-fast decomposition ([24] and references therein). We first focus on the initial phase of the burst (e.g. pink star, Figure 4A). For now, *E_K_* and *I_pump_* are regarded as fixed, i.e. as constants taking their (arbitrarily chosen) values at the pink star (−83.17 mV,14.96 µA/cm^2^). We pick a value of *E_Na_*, run a simulation, wait until transients die out, and measure the minimum and maximum values of *V_m_*. This procedure is repeated for the range of *E_Na_* values on the abscissa of Figure 5B. On the ordinate we plot the corresponding minima and maxima of *V_m_*. A striking feature is that in certain regions of the diagram there are more than two ordinate values; this is a hallmark of bistability as discussed further below. Since *E_Na_* = 40.2 mV at the pink star, the diagram demonstrates that the CLS injury places the system in a superthreshold oscillation (i.e. spiking) regime. The spiking trajectory at the pink star in Figure 4A and 5A corresponds to the stable limit cycle (i.e., stable periodic oscillation) in Figure 5B.

At the onset of firing (pink star in Figure 5A), the pumps are still driving *E_Na_* upwards and past (i.e. to the right of) the Hopf bifurcation point (see Figure 5B). In that region, the fixed point, around which the STO oscillates, has become unstable; the trajectory moves towards the only possible stable state, a stable limit cycle corresponding to repetitive firing (APs).

During firing *E_Na_* is decreasing and consequently this limit cycle shifts to the left and approaches the bistability regime. During this process, both amplitude and frequency of the limit cycle decrease. In the bistability region of Figure 5B, the system has two stable states: a steady fixed point or resting potential (i.e. RP, marked by solid line) and a superthreshold oscillation (i.e. APs, or tonic firing). Between these states is an unstable limit cycle. The system phase space is then split into two sets of points. In the first set, typically near the RP, phase space solutions converge to the RP. In the other, typically further from the RP, solutions converge to the tonically firing solution. In both these sets, initial conditions close to the unstable limit cycle yield solutions that diverge away from it in an oscillatory manner: in the first set the oscillation grows into the tonic solution, and below, oscillations damp out towards the RP. Further, a perturbation from the RP must “jump over” this unstable limit cycle in order to lead to tonic firing, and vice-versa.

Near the offset of firing (green dot), with *E_Na_* still decreasing, the dynamics move toward a region of period doubling (PD). Sometimes the trajectory falls off the limit cycle on the upper branch before reaching this region such as in Figure 5C. Other times during the same solution the trajectory enters briefly into the period doubling regime. This manifests itself as a longer interspike interval at the end of the firing phase (see Figure 4A, second burst). For parameter choices corresponding to bursting the trajectories are not periodic, i.e. they appear to be chaotic unless the transients exceed our simulation times. The precise dynamics in this region warrant more extensive investigation beyond the scope of this paper. Note also that the AP amplitude decreases together with *E_Na_*. This is expected as the ion concentration gradients drive the action potential. This suggests that the upper branch of the PD regime is not reached by the trajectories near firing offset.

Let us inspect Figure 5 in more detail. Figure 5A shows that both *E_Na_* and *E_K_* are dynamic, changing slowly during a bursting period. Figures 5B and 5C capture the behavior of the system near the two transitions (pink star and green dot) in Figure 5A. The pink bistability region in Figure 5B – corresponding to the pink star – sits between a limit point (LP: where a “saddle-node of limit cycles” bifurcation occurs), and a Hopf bifurcation point (HB: where a transition between a stable fixed point and a stable limit cycle occurs). The green region in Figure 5C lies between (at left) an area (PD) of complex dynamics where many period-doubling bifurcations occur and (at right) a HB. The unstable limit cycle solution (marked by open dots) between the stable fixed point and stable limit cycle is the aforementioned threshold boundary. The arrow in Figure 5B shows the direction in which *E_Na_* changes during the firing phase (*E_K_* changes as well). Likewise, the blue arrow in Figure 5C shows how *E_Na_* changes during the STO phase. During the bursting period, the bifurcation diagram in Figure 5B slowly morphs into the Figure 5C diagram and back. These two diagrams, snapshots of the system's possible steady state behaviors at the two ends of the bursting cycle, help us understand how the whole solution plays out during an entire bursting cycle.

Interestingly, from Figure 5B one might think spiking would continue until the point LP is reached, where *E_Na_* is almost nil. However, the pump's dependence on [*Ion*]s limits this decay, such that a PD region appears and truncates the spiking phase at a relatively large value of *E_Na_* (note the difference in *E_Na_* scales in Figure 5B and 5C).

The two-parameter phase diagram in Figure 5D provides a view of the burst dynamics from the *E_Ion_* perspective. Here the pink star and green dot have the same meaning as in Figure 5A–C (see caption). The black loop connecting pink star to green dot is the projection of the 3D trajectory in Figure 5A onto the *E_Na_*−*E_K_* plane, and displays the *E_Na_* and *E_K_* trajectories during bursting. The solid and dashed curves are, respectively, sets of all the LP and HB points for the range of *E_Ion_* of interest (exemplars of these points were seen in Figure 5B,C). The area bounded by them is the bistability regime. This involves computation of many bifurcation diagrams along the entire bursting trajectory, choosing from the trajectory the two extremal pump values (pink for the lowest and green for the highest). One can imagine that, when the system state moves from pink star to green dot, the periodically varying *I_pump_* pushes the bistability regime between the pink-curve-bounded and green-curve-bounded areas. Thus, injured node dynamics switch between different modes, resulting in bursting behavior.

### Nav-CLS phase diagram: 3 *g_Na_* subpopulations

**Figure 6:**
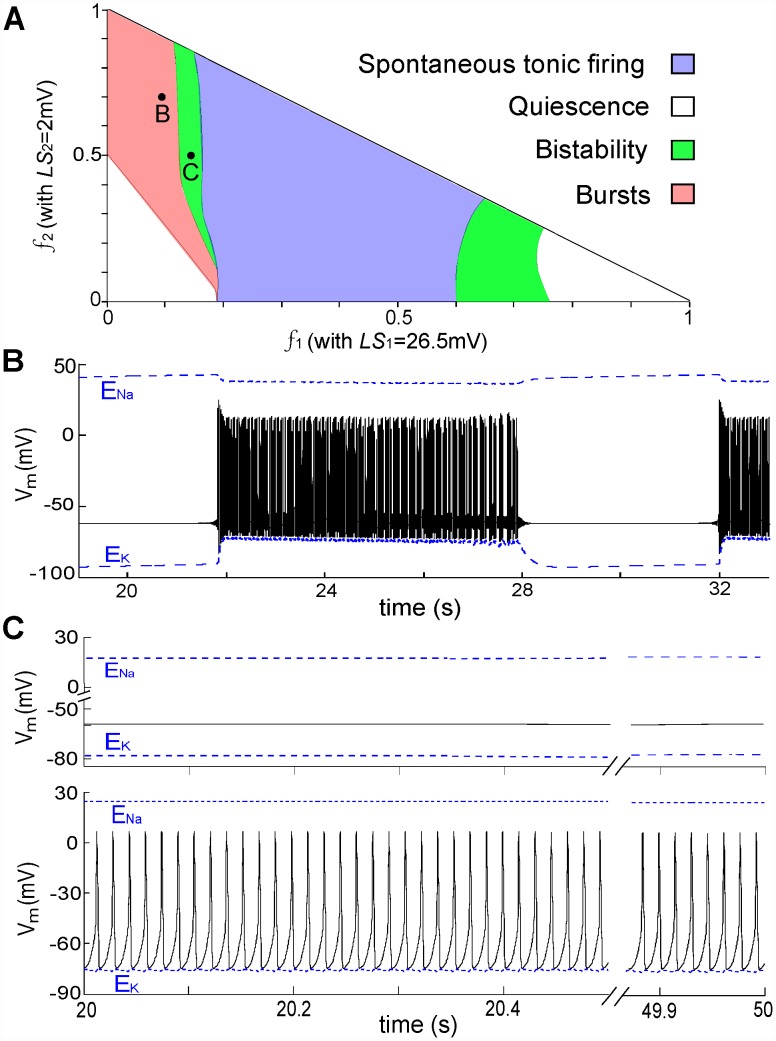
Classes of Nav-CLS-induced activities with two injured and one intact *g_Na_* population. (**A**) Membrane excitability map of injured node with *LS_i_* = [26.5,2,0]mV. White, pink, green and blue regions represent different stable state activities: quiescence, bursts, tonic firing, and bistability between quiescence and tonic firing. Here the fraction of intact *g_Na_* is *f_3_* = 1−*f_1_−f_2_*. (**B**) and (**C**), typical *V_m_* trajectories when the system is in bursting and bistability regimes, respectively.

Various spontaneous activities of an injured node with three *g_Na_* subpopulations (*LS_i_* = [26.5,2,0]mV) comprising different fractions are presented in Figure 6A. The fractions (*f_1_* and *f_2_*) corresponding to the Nav-CLS populations form the *x*- and *y*- axes; the fraction of intact channels is simply *f_3_ = 1−f_1_−f_2_*. When the percentage of channels with deeper injury (i.e. *LS_i_* = 26.5 mV) increases, the state of the injured node goes from RP→bursting→bistability (APs or RP, depending on the initial condition)→ tonic firing→bistability→RP. For two random points from the burst and bistability regimes, *V_m_* is plotted in Figures 6B and 6C, respectively. Overall, STOs can arise from various combinations involving decay to or growth away from RP, but the particular behavior seen depends sensitively on the percentage and degree of injury.

### Nav-CLS and susceptibility to noise-induced bursts

**Figure 7:**
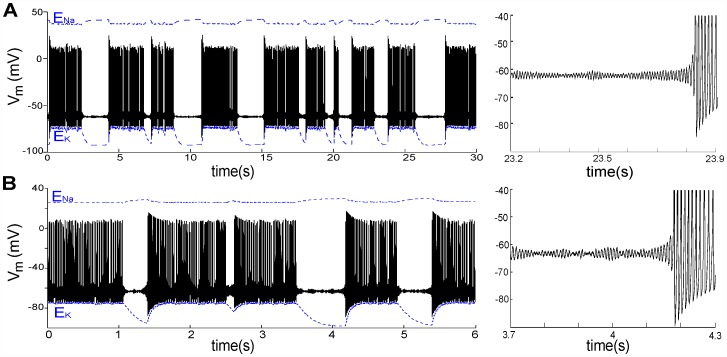
Noise induces or modifies bursting. (**A**) Without noise, bursts are as shown in Figure 6B but here, under the influence of noise (σ = 0.1 µA/cm^2^), the bursting periods are random. Right (here and in B): an expanded section showing STOs induced by noise via coherence resonance. (**B**) Without noise the system is in the bistability regime as shown in Figure 6C, but here, noise (σ = 0.4 µA/cm^2^) causes *V_m_* to randomly visit quiescent or repetitive firing states, hence producing noisy bursts.

Neuronal noise arises naturally from a wide variety of sources including channel noise, synaptic noise, electrogenic ion pumps, and thermal noise. In this subsection we investigate the influence of current noise on spontaneous electric activity of a node with Nav-CLS injury by including a simplified Gaussian white noise σξ on the right side of Eq 1 as an additive term. This additive noise ξ has zero mean, and its autocorrelation function is <ξ(t)ξ(s)> = δ(t−s) where δ(·) is the Dirac delta function; σ is the noise strength (in µA/cm^2^). For a node, the white noise can be taken as describing conductance and thermal noise. By definition it has equal power at all time scales, in particular beyond the fastest time scales present in our deterministic (i.e. noiseless) node model. Furthermore, because the central limit theorem applies to the sum of different independent fluctuation sources acting on *V_m_*, its amplitude distribution is assumed to be Gaussian (see for instance, [46]). For a deterministic bursting activity with burst period BP = 10.2 s illustrated in Figure 6B, additive noise dramatically decreases BP (Figure 7A). It also makes BP irregular because the crossing of the threshold by the *V_m_* trajectory is now a probabilistic process, especially in the vicinity of the threshold [47].

When the node operates in a bistable regime, as in Figure 6C, this additive noise causes stochastic switching between the quiescent and tonic firing modes (Figure 7B); noise-induced bursts appear. In fact, the injured node produces bursting in both the burst and bistability regimes of Figure 6A. With higher noise intensity, the mean BPs of noise-induced bursts is shorter. Such bursting is also referred to as noise-induced mixed-mode oscillations [48]. This phenomenon has been found in experimental settings, for example the bistable squid giant axon [49], electroreceptors of paddlefish [50] and entorhinal cortex neurons [51], as well as in excitable neuron models such as the Hodgkin-Huxley model [49], the FitzHugh-Nagumo model [48], and an excitable spine model [52].

The expanded sections in the right column of Figure 7 demonstrate that, upon introduction of noise, the silent phase of the burst behaviour in Figure 6B and the steady state in Figure 6C turn into STOs. This is observed in injured primary sensory neurons [53], and constitutes coherence resonance (CR) (or autonomous stochastic resonance) wherein noise alone excites subthreshold oscillatory responses in excitable systems (see [47], [54] and references therein). Noise continually modifies the firing pattern because it randomly pushes the system into and out of different regions of phase space, - similar behavior has been described for thermoreceptors [46]. In terms of Figure 5C, when the system is on the lower STO branch, the noise induces the behavior seen at the nearby Hopf bifurcation. In summary, for our Nav-CLS system, noise can elicit a random mixture of the behaviors seen in the phase diagram in Figure 6.

### Nav-CLS variance and diverse patterns of ectopic excitation

**Figure 8:**
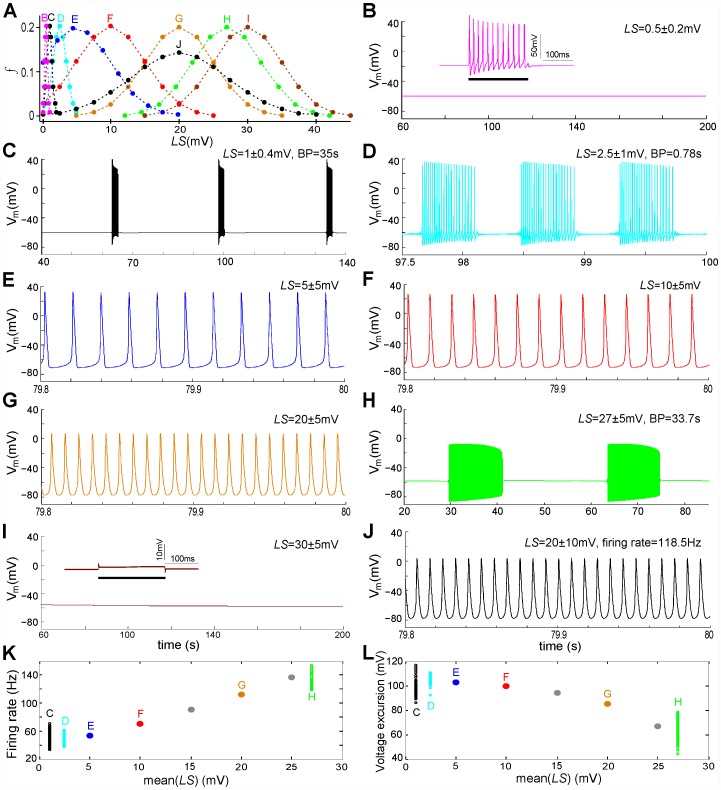
Classes of Nav-CLS-induced activities. (**A**) Nav-CLS of Gaussian distributions with mean±SD(*LS*) = [0.5±0.2, 1±0.4, 2.5±1.0, 5±5, 10±5, 20±5, 27±5, 30±5, 20±10]mV. (**B**)–(**I**) various activities of a node are demonstrated for increased injury (i.e. > mean(*LS*)): quiescence (B), burst (C,D), tonic firing (E,F,G), burst (H) and quiescence (I). Insets in (B) and (I) show the effect of injecting a 12 µA/cm^2^ stimulating current. (**J**) Doubling the SD(*LS*) in G from 5 to 10 mV slightly increases the tonic firing rate. (**K**) Instantaneous firing rate (inverse of the interval between two spikes) for tonic or within-burst spikes, versus mean(*LS*), corresponding to firing patterns C–H (see color coding). (**L**) Spike (tonic or intraburst) voltage excursions begin to decrease for mean(*LS*) >10 mV.

Next, we explored the spontaneous activity of an injured node with different Nav-CLS variances. Gaussian distributions of Nav-CLS were imposed with different means and standard deviations (SDs). As illustrated in Figure 8A, we tested mean(*LS*) = [0.5,1,2.5,5,10,20,27,30,20]mV with corresponding SD(*LS*) = [0.2,0.4,5,5,5,5,5,5,10]mV. With increased injury intensity (greater mean(*LS*)), the *V_m_* changes are dramatic (Figure 8). There is a progression from quiescence (B)→bursting (C–D)→tonic firing (E–G)→bursting (H)→quiescence (I). For a smeared Nav-CLS injury as small as *LS* = 1±0.5 mV, the node generates bursts (BP = 35 s), indicating this system's extreme susceptibility to even small changes in window current. (Left-shifting Kv activation by an equivalent amount [11] does, however, provide some stabilizing protection for *V_m_*). A slightly larger trauma (mean(*LS*) = 2.5 mV) shrinks BP dramatically to 0.78 s, then further increasing mean(*LS*) leads to tonic firing. If, however, mean(*LS*) is taken to 27 mV (Figure 8H), bursting with STOs (not visible) returns. Then, for mean(*LS*)≥30 mV no spiking occurs. In Figures 8C, D, H the within-burst firing rate and *V_m_* excursions decrease with time. In Figures 8E–G the instantaneous within-burst and tonic firing rates gradually increase with mean(*LS*) (Figure 8K). The corresponding mean *V_m_* excursions decrease with increased mean(*LS*) (Figure 8L). With a smear of Nav-CLS that encompasses a wider range of LS values (SD = 10 mV: Figure 8J; see Figure 8A)), the injured node fires tonically, as in the lower SD case for the same mean(*LS*) (Figure 8G). Evidently the larger *LS* components of the SD = 10 mV distribution do not sufficiently dominate to yield the type of quiescence associated with a (smaller SD) 30 mV *LS* (Figure 8I).

### Effects of varying maximal pump current

**Figure 9:**
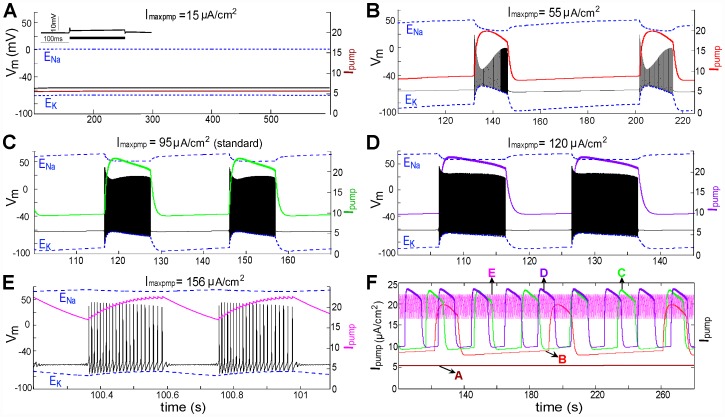
Increased *I_maxpump_* shortens bursting periods (BP) and burst duration (BD). (**A**)–(**E**) Five values of *I_maxpump_* as labeled elicited activities as shown with *LS*: Gaussian distribution, 2.5±1 mV (steady-state values in the absence of CLS injury are listed in Table 2). Note the different time scales in each panel. For a bursting node, *I_pump_* is periodic with a period equal to BP. Inset in (A) shows the effect of injecting a 12 µA/cm^2^ stimulating current. (**F**) For direct comparison of BPs and burst durations, *I_pump_* traces from panels **A–E** (color coding preserved) are plotted together.

In Figure 3D we saw that decreased *I_maxpump_* suppresses spontaneous tonic firing with CLS injury. In Figure 9 we look at the effect of varying the pump activity on bursting patterns in a mildly injured node (*LS* = 2.5±0.5 mV). *I_maxpump_* = 95 µA/cm^2^ (the value used in Figure 9C) is our standard or healthy cell reference. *I_maxpump_* depends on the density of pump expression in the membrane, which will differ among axons and also for different regions of a given axon. It also varies with a multitude of factors, including injury conditions that affect the turnover rate at individual pump molecules. To explore these possibilities in a mildly injured node, we varied *I_maxpump_* over a wide range below and above the standard value in Figure 9C.

Figure 9B shows that a lower *I_maxpump_* is associated with longer interburst intervals (IBI: see Figure 4) than for the standard case (Figure 9C). Figure 9A, an extreme example in which excitability is lost, has an infinite IBI. Increasing *I_maxpump_*, by contrast, initiates burst of spikes and shortens both burst duration (BD) and burst period (BP) (Figure 9D,E), reflecting the hyperpolarization produced by the electrogenic Na/K pump. The differences between Figure 9B and 9C are consistent with experimental observations in rat spinal interneurons where, during the course of pump blockade, the burst rate decreases (larger BP) [42]. It is noteworthy that for the uninjured system, varying maximal pump current had little impact on the steady- state *V_m_* (see Table 2). What vary substantially are the steady state *E_Ion_*. This is consistent with the mechanistic interpretation that *I_pump_* oscillations responding to chronic Nav-CLS-based Nav-leak underlie the array of bursting patterns in the many simulations above. The basic phenomena (ectopic APs and STOs) are extremely robust properties of nodal membranes with mild Nav-CLS and a pump, and appear in various manifestations without the need to invoke novel conductances or Nav channels with peculiar kinetics.

**Table 2:** Steady-states for intact nodes (*V_m_* = −59.9 mV) as *I_maxpump_* varies (units as in Table 1).

Fig. 9	*I_maxpump_*	*E_Na_*	*E_K_*	*I_pump_*
**A**	15	3.5	−73.7	5.1
**B**	55	42.8	−82.3	8.3
**C**	95	54.3	−84.6	9.2
**D**	120	58.6	−85.4	9.6
**E**	156	63.1	−86.3	9.9

### Nav-CLS, pseudo-first order activation and subthreshold persistent current

**Figure 10:**
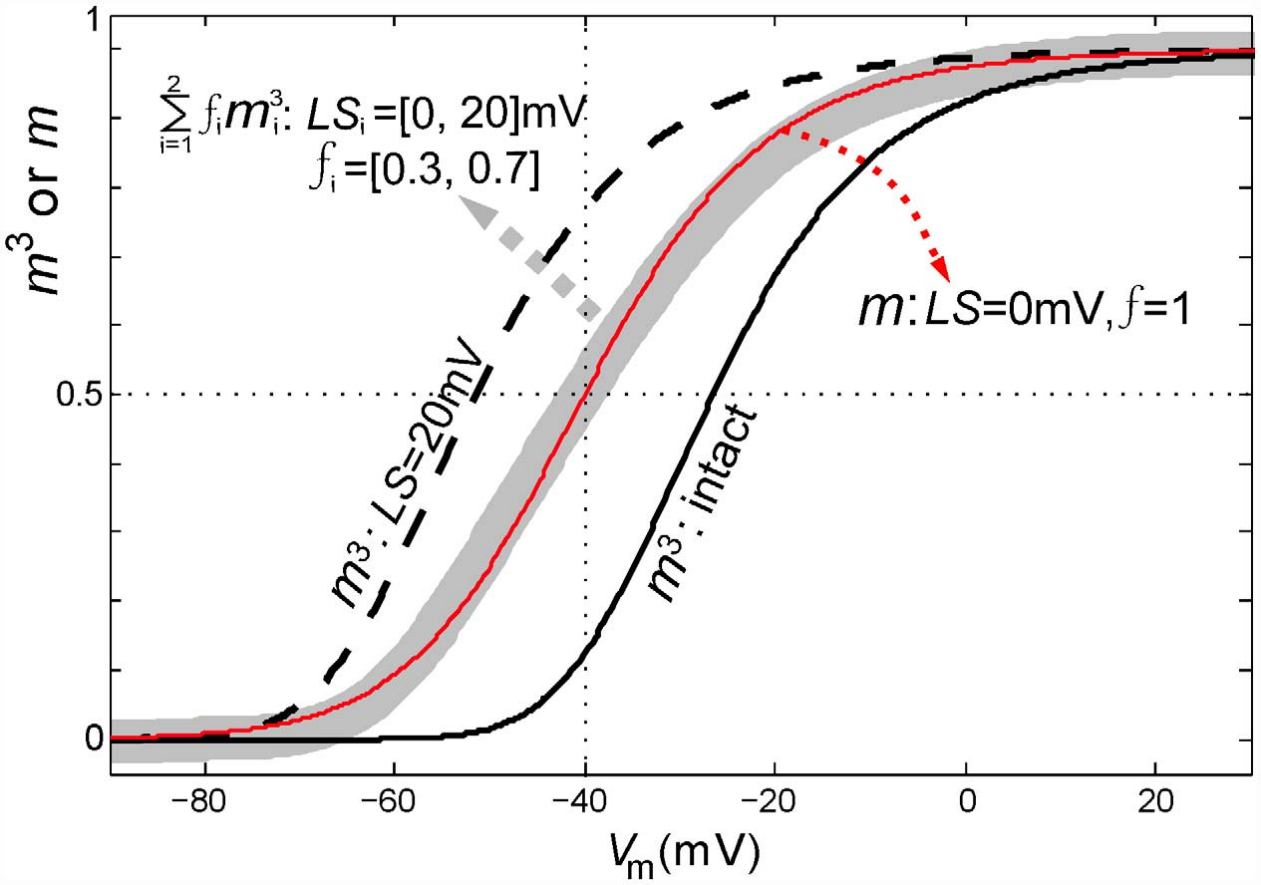
Partial Nav-CLS in a population and apparently first order activation kinetics. Equilibrium activation values (*m^3^*, 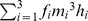 and *m* ) of co-existing intact Nav and CLS Nav channels plotted as a function of *V_m_*. Black solid and black dashed line represent *m^3^* for intact and 20 mV CLS channels with *f* = 1. For a membrane with large Nav-CLS injury given by: *LS_i_* = [0,20]mV and *f_i_* = [0.3,0.7], the gray bold line denotes 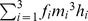. Red line: *m* of the intact channels.

Unlike nodes, somata express multiple Nav isoforms, and in recent modeling work of injured somata, Kovalsky *et al* [21] showed that a system with three distinctive *g_Na_* sub-types (but no Kv and no Na/K pumps) yields STOs, provided the two slow *g_Na_* sub-types are given 1st rather than 3rd order activation. This provided the system with the “several delayed components” needed to mimic the repertoire of STO-triggered phenomena of live neurons. To simulate Nav1.7 and Nav1.8 interacting in a fashion that elicits STOs, Choi and Waxman [23] also depicted one of the *g_Na_* sub-types with 1st order activation. Interestingly, in our system, left-shifting a fraction of the *m^3^ h*-based *I_window_* acts like introducing a lower order activation process (see Figure 10). What physical change in injured axons that could yield such pseudo-first order *m_∞_(V)* curves for total *g_Na_*, even though individual channels continue to exhibit their normal *m^3^* activation? Heterogeneity in the extent of bilayer damage could cause this by fluidizing a fraction of the nodal bilayer. This would yield a subthreshold persistent *I_Na_*. Differences in bilayer packing in different parts of the membrane [3], [36] also arise developmentally in some circumstances. In a given region of plasma membrane, a membrane remodeling process that places some Nav channels in (say) cholesterol-poor [55] and others in cholesterol-rich subdomains could, in principle, create or “tune” physiologically useful subthreshold persistent currents.

## Discussion

Here we demonstrated that different patterns of spontaneous activity can be expected from mildly damaged nodes of Ranvier in which some or all the Hodgkin-Huxley type Nav channels have undergone a coupled left-shift in their activation and inactivation kinetics. This is called Nav-CLS [11] and as a form of Nav-leak, could result in the ectopic activity that emanates from injured nodes. As detailed in the *Introduction*, there is growing evidence that activation and inactivation are left-shifted in various chemical and mechanical injuries in different cell types and Nav isoforms [10], [12]–[19]. In more focused studies, it has been demonstrated that these left-shifts are coupled [8]–[10], and this CLS in Nav1.6 (the nodal isoform) is the basis of our study [11]. In diverse sick excitable cells, it has been hypothesized that Nav-CLS occurs wherever channel-embedding bilayer becomes structurally degraded and fluidized as a result of mechanical or chemical insults [3], [4].

The following are the main findings of this study which is, essentially, a dynamical analysis of a standard node of Ranvier model with Na/K pumps, under conditions of coupled-left-shift (CLS) injury to some or all of the Nav channels and injury to the Na/K pump in terms of lowered output. The analysis uses simulations and bifurcation theory.

1. With *E_Ion_* values fixed (i.e. pump activity irrelevant) at standard or mildly depleted values (10–15%), *LS* injury levels up to 20 mV (including for “smeared”, i.e., Gaussian distributions of CLS) elicit either quiescence or tonic firing. With increasing *LS*, a transition from quiescence to tonic firing occurs via a subcritical Hopf bifurcation, then at yet higher LS, tonic firing is replaced by quiescence via another subcritical Hopf bifurcation. If *E_Na_* and *E_K_* both approach zero, that bifurcation is supercritical; in its vicinity, tonic APs of arbitrarily small amplitude occur.
2. Maximal pump current is an important determinant of nodal excitability. When increased above a certain value, it yields an abrupt transition from quiescence to a relatively high rate of tonic firing; this is a supercritical Hopf bifurcation.
3. There exist CLS injury conditions where the system is bistable, exhibiting either quiescence or tonic firing. In this bistability regime, behavior depends on initial conditions in a complicated manner not explored here. Stimuli, like spikes arriving from an upstream node, could thus toggle the system these two behaviors.
4. Spontaneous bursting activities occur for small and for large *LS* injuries, while intermediate intensity injuries elicit tonic firing. In the case of bursting, time-varying *E_Ion_* values due to pump activit*y* provide a slow almost periodic element in the system; *E_Ion_* values approach zero during the burst and grow during the interburst interval. Slow-fast decomposition of the full dynamics reveals that these slow *E_Ion_* bring the fast spiking dynamics into and out of a limit cycle (tonic firing) regime.
5. STOs can appear with single injured Nav population or for complex CLS injuries. STOs that bracket bursts of firing are associated with the complex eigenvalues governing dynamics in the vicinity of the lower branch of fixed point equilibria. Burst onset is associated with a subcritical Hopf bifurcation while offset is organized around a saddle-node bifurcation of limit cycles. Period-doubled solutions exist near this latter bifurcation, and the limit cycle can collide with these solutions, producing abrupt changes in the interval between successive spikes. Bursting solutions tend not to repeat over many bursts, and thus appear to be chaotic (not analyzed in detail).
6. Including small levels of Gaussian white noise in simulations causes burst characteristics to fluctuate and yields more long-lived STOs, consistent with the reported propensity of injured axons to exhibit STO phenomena.
7. Inhomogeneous CLS is sufficient to explain a rich variety of axon excitability behaviors. Combinations of CLS-injured Nav channels, all exhibiting realistic *m^3^* activation behavior, yield flattened *g_Na_*(*V*) curves similar to those of other models that, of necessity, include at least one *g_Na_* with *m^1^* activation.

### Nav-CLS and pathophysiological Nav leak

Regions of membrane with mild Nav-CLS have a “subthreshold” persistent *g_Na_* (a Nav-leak conductance) due to *g_window_* operating abnormally close to the cell's normal resting *V_m_*; when only a small fraction of Nav channels are affected, the cell's *g_window_* plot has a shoulder as in Figure 4C; such plots strongly resemble coupling-induced subthreshold persistent conductance of pacemaker neurons [56]. Like that physiological pacemaker, our injury model includes no slow mode (or non-inactivating) *g_Na_*. Calculations [3] show, in any case, that at voltages near typical AP thresholds, left-shifted *I_window_* would dominate over equivalently left-shifted non-inactivating *I_Na_*. In much of this study, we mimicked “smeared” extents of bilayer damage via Gaussian distributions of Nav-CLS, reflecting the notion that nodal axolemmal injury is unlikely to exhibit a unique intensity. Smear, though expected in situ [2], [8], is not actually required for neuropathic activity. Use of assorted smears confirmed that CLS-fuzziness in no way abrogates dysfunctionality and it demonstrated the robustness of Nav-CLS as a model for leak. Nav-CLS injury thus provides a minimalist explanation for multiple spontaneous modes of firing in which APs, bursts and subthreshold oscillations (STOs) occur in various patternings. In severe cases, it also predicts depolarizing block [11]. We used bistability diagrams and various bifurcation analyses to help clarify the mechanistic underpinnings of the Nav-CLS-induced dynamics.

A mildly injured excitable cell cannot survive for hours or days (the time course, e.g., of diffuse axonal injury) without operational Na/K pumps. In electrical models of axon injury, pumps do not only maintain ion gradients, they also contribute to slowly varying excitability patterns of the damaged-but-viable axons [11], [29], [30]. Pursuing this further here, we found the following: mild Nav-CLS induces STOs through a gradual reduction of firing threshold mediated by slow *E_Ion_* dynamics whose characteristic times depend on pump-mediated fluctuations of extra- and intracellular ion levels. Threshold voltages for firing are not fixed but depend on *E_Ion_*. *E_Ion_* fluctuations induced by Nav-CLS produced, in a bistable fashion, both STOs and bursts of full-sized APs. With mild Nav-CLS injuries, adding Gaussian white noise to the current-balance equation induced erratic bursting, or, where injury was already causing sustained bursting, noise influenced the rates and durations of the ectopic signals. These effects of noise are consistent with those seen in a recent computational study of neuropathic firing in a hyper-excitable system with more ion channel species than used here, but without pumps [57]. In the following we discuss and provide more context for the above results.

### Problematic rhythmicity patterns associated with mild axonal injury

Trauma-induced [58] and ischemia-induced [59] Nav leak triggers Ca^2+^-excitotoxicity, the proximate cause, in diffuse axonal injury and oxygen glucose deprivation, of axon demise. A mechanistic understanding of early stages of Nav channel dysfunction, when salvage is possible [60], is needed. Although pathological TTX-sensitive *Na^+^* leaks are attributed to increased “persistent *I_Na_*” (e.g. [61], [62]) or slow-inactivating *I_Na_* [63] it is not known how membrane damage elicits chronic Nav-leaks. A molecular-level understanding is lacking as to what constitutes leaky native Nav channels. A common Nav-leak culprit in diverse sick excitable cell conditions is, we propose, Nav-CLS [3]. For recombinant Nav1.6, this phenomenon occurs as an immediate consequence of mechanical membrane damage [8], [11]. Any fraction of *I_window_* (an attribute of fast *g_Na_*) that has become left-shifted acts as a leak [3], [8] that could trigger positive feedback excitation.

In previous modeling [11], we established that mild axonal Nav-CLS would effectively dissipate [*Na^+^*] gradients, bringing on excitotoxicity. Total nodal *g_Na_* was modeled by an intact fraction plus a second fraction with a discrete level of CLS. Various “Nav-CLS injuries” were imposed, *V_m_ (t)* was plotted as new steady-states were attained, then excitability was probed with current injection. Mild Nav-CLS caused spontaneous APs (tonic or damped trains). With Na/K pump activity included, it caused AP bursts whose specifics varied with compartmental volumes. From temporal patterns of [*Na^+^*] gradient rundown, it was evident that Nav-CLS type injuries could cause axon demise by overwhelming an axon's capacity for ion homeostasis. Injured nodes, moreover, generated pernicious ectopic signals and impaired transmission fidelity during saltatory propagation. Pathological activity patterns, plotted as a function of the extent (in mV) of Nav-CLS, and of the fraction (0–1) of affected nodal Nav channels, fell into several regimes.

What can we infer about responses to stimulating inputs when there is Nav-CLS injury? Effects of stimulation, a multi-faceted issue, was addressed for nodes with fixed reversal potentials in a preliminary study [11] involving two Nav populations, one intact and one with a specified CLS injury. Spontaneous firing occurs over a range of damage, then beyond a critical threshold level (*LS_c_*) the system is quiescent. Stimulating with constant current yields a larger maximal firing rate but, due to depolarizing block, quiescence occurs with less damage (*LS<LS_c_*). Effects of stimulation are currently under study for bursting nodes (model with pumps as in Figure 5). Because bursting is a globally attracting behaviour, a single spike as a form of input may transiently change a bursting cycle but not its steady state. For the regime we considered, bifurcation analysis reveals that a single spike cannot “trigger” bursting behavior.

### Nav-CLS and neuropathic pain

Though ectopic signals in vivo are generated at both axons and somata [64], peripheral neuropathic (as distinct from nociceptive) pain mechanisms are mostly studied via ectopic activity in damaged somata such as those of primary mechanoreceptor afferents [62], [65], [66]. A closely-related issue of relevance here is that of low threshold afferents, dubbed “algoneurons” [67]. These are non-pain sensory neurons that can serve double functions, signalling pain only when peripherally hypersensitized due to trauma, inflammation and the like. Spontaneous activity patterns described here are like those of both damaged mechano-afferents neurons and hypersensitized algoneurons. This is a post hoc assessment; we monitored behaviors across parameter space, but did not adjust parameters to mimic particular neuron activities. Clearly, it would be interesting to determine if “hypersensitization” in algoneurons corresponds to Nav-CLS in damaged peripheral endings.

### Mechanisms for generating STOs

Emerging from that “injury”-modified Hodgkin-Huxley axon (two *g_Na_* sub-populations and the Na/K pump) was a critical feature of the electrical dysfunction of injured excitable membranes, abnormal STO phenomena [11]. We lacked, however, a dynamical understanding of the phenomenon and its robustness; given the prevalence of STOs in neuropathic firing (plus physiological correlates) this represented a significant gap. Typically, STOs and injury have been modeled with non-inactivating and/or slow-gating *g_Na_*. Our model - Nav-CLS - includes only a fast-gating *g_Na_* to which a simple biophysically-justified (and biophysically-explicable) modification has been applied at varying intensities. Here, using smeared-intensity versions of Nav-CLS to model injury, we generated diverse STO phenomena.

The “spared but impaired axons” of injured tissue are targets for Nav antagonists (see [21]) and such mildly-injured axons were our focus here. We examined sustained spontaneous (ectopic) behaviors, extending our analysis first to three *g_Na_* sub-populations (i.e., for otherwise identical *g_Na_* mechanisms, the Nav population was given three extents of Nav-CLS), then to various Gaussian-distributed (“smeared”) Nav-CLS populations. Realistically untidy, but conceptually simple, all these Nav-CLS injury variants exhibited the key components of the injury repertoire. STOs arise with (Figure 8D) or without smear. Along with the basic spontaneous activities (APs and bursts), injured nodes spontaneously produced sustained STOs (Figure 3) and STOs as parts of bursts consisting of clusters of spikes and STOs (Figure 4).

### Pump/CLS-leak: a slow “component” of an oscillatory system

Na/K pump turnover rates are sensitive to substrate concentrations, membrane potential [68] and lipid packing [39], [40]. Quiescent (“at rest”) axons maintain a steady basal pump activity that counteracts all system leaks. In our model *I_pump_* is always substantially smaller than *I_Na_* and *I_K_* (∼22-fold in Figure 4A) and pump rates oscillate significantly more slowly than APs (e.g., Figures 8C,D, ∼2 orders of magnitude slower). In our simulations of injury, small amplitude slowly oscillating *I_pump_* plays an essential role in mediating spontaneous *V_m_* excursions (Figures 3, 4 and 9). Larger magnitudes for *I_maxpump_* (see Figure 3D and Figure 9) accelerate the efflux/influx of Na/K ions. This was associated with dynamical changes, from RP to STOs to bursts or tonic APs. Figure 3D in particular is a bifurcation diagram; it characterizes the transition from quiescence to tonic firing as *I_maxpump_* increases. This shows clearly the increase in the amplitude of voltage excursions *V_m_* and the decrease in firing rate. Figure 9 shows the effect of *I_maxpump_* in the bursting regime. As *I_maxpump_* increases, we go from quiescence to bursting. All characteristics of the burst summarized in Figure 4 shorten with this increase.

In the injured nodes, therefore, a reduced *I_maxpump_* (due, say, to ATP depletion and/or to a more fluidized pump-embedding bilayer) would modify ectopic excitation patterns. In line with this, in endogenously bursting snail neurons, inhibiting the pump shortens burst periods and increases spike frequency [69], though unlike injured nodes, pacemaker neurons have multiple slow processes that confer resilience on specific oscillatory patterns. Dynamic transitions of *I_pump_* are seen in a number of rhythmically active cells including sinoatrial node cells [44] and trigeminal motoneurons [70].

Insofar as Na/K pumps show greater ATPase activity in membranes with greater lipid packing order [39], [40], membrane injury that caused Nav-CLS should simultaneously impair pump activity. To better understand ectopia arising from injured nodes or axon initial segments, it would be helpful to know if Na/K pump inhibitors (e.g. ouabain, but also generic membrane fluidizers [43], [71]) reduce ectopic burst firing in mildly damaged axons. In this regard, we note that peripherally-applied ethanol (shown recently to inhibit neuronal Na/K–ATPase [72]) induces a “feeling no pain” condition when injected locally to relieve peripheral trigeminal neuralgia [73].

### Bifurcation analysis: Nav-CLS/pump STOs help trigger spikes by decreasing firing threshold

Although neuropathic STOs trigger APs in many neurons [58], [74], STOs are not principally a neuropathic phenomenon. In the CNS [75], STOs are a filtering mechanism by which neurons decode oscillatory inputs from other neurons in a frequency dependent manner [76]. Disparate underlying mechanisms for STOs have been noted, including Ca channel *I_window_* [77] and HCN-channel based *I_h_* [75], and, of particular interest here, a fast *g_Na_*-based [56] subthreshold persistent *I_Na_* [78]). Our mild Nav-CLS-based *I_window_* resembles that mechanism, but generates STOs and AP bursts only in conjunction with the electrogenic pump. STOs arising spontaneously at nodes are, by definition, pathologic, and given the dominance of nodal conductances by *g_Na_* (specifically, Nav1.6 channels) would likely be simpler in nature than the STOs elicited (by current stimulation) in multi-Nav isoform sensory neuron somata [21] or in double-Nav nociceptive terminals [23].

STOs simulated here involve a small-amplitude rhythmic mechanism that develops with mild Nav-CLS *I_window_* (Figures 3 and 4) operating near what would normally be the RP level. Bifurcation analysis of this system enables us to propose that slow dynamics of the firing threshold are responsible for the timing of spikes. In a system where the *E_Ion_* vary, the firing threshold varies too, gradually decreasing in the interval between successive STOs (Figure 9). This is broadly consistent with experimental findings of a decreased spike rheobase in injured dorsal root ganglion neurons [79] and, more general terms, seems consistent with the injury-induced hypersensitization of algoneurons [67]. Models involving fixed *E_Ion_* cannot exhibit spontaneous oscillations of firing threshold, but in vivo, and especially in fine processes (e.g., Nav-rich nociceptive endings), fluctuating [*Ion*]s and pump currents are expected once channels activate.

### Leak/pump counterplay and the oscillating threshold: implications for healthy membranes

Analyses of the electrical excursion diversity seen in excitable cells gradually going through their death throes have, historically, been fruitful in providing sketches of the behavioral diversity of healthy excitable cells. For example, the Hodgkin [80] classification of various spiking behaviors in isolated crayfish axons and the Morris-Lecar [81] analysis of giant barnacle myocyte voltage oscillations provide the springboard for analytical descriptions of oscillatory types in mammalian interneurons [82]. Here we simulated sick cell behaviors in a context where large fluxes into small volumes made it crucial to include the contributions of the electrogenic Na/K pump. From this emerged an STO mechanism that triggers bursts of spikes in a manner not, to our knowledge, previously recognized, i.e., the oscillating threshold. Since threshold occurs at *V_m_* values where fluxes unbalance, threshold varies with *E_Ion_* variations. An ultra-simple biological exemplar of this point is a *g_Ca_*-based biphasic AP (Figure 3c of [83]) that shows different thresholds for its depolarizing and hyperpolarizing excursions and that is modeled simply by letting [Ca^2+^]_intracellular_ accumulate with *g_Ca_* activation (Figure 3d and Equation (8) of [81]).

Here we explicitly considered injury-induced Nav-CLS in a setting where Nav1.6 would be the exclusive Nav isoform. But Nav-CLS may have resonances for healthy excitable membranes with two or more Nav isoforms with different voltage midpoints, such as seen in axon initial segments [33] and nociceptive nerve endings [23]. Moreover, even with a single isoform, non-homogeneous physiological modulation (e.g., from inhomogeneous lipid packing density) could yield CLS-like situations that might affect tuning (e.g,. in the cochlea [83], [84]). As in vivo lipid imaging becomes refined, it may be possible to detect lipid-packing changes before and after injurious stimuli at nodes or axon initial segments. This is important given that the activities of voltage-gated channels and the Na/K pump are both modulated by lipid packing [37], [39]. The kinetic phenotypes of the membrane proteins simulated here are, of course, constrained by amino acid sequence, but they also vary according to the bilayer mechanical milieu. Our work here suggests how, in the face of bilayer injury, the sensitivity of Nav channels to bilayer mechanics can be perilous. By the same token, it may spell opportunity, with developmentally controlled CLS-type kinetic modulations spawning behavioral amplifications beyond what would initially be expected from a limited set of membrane proteins.

## Methods

### Node of Ranvier with variable amounts of membrane damage

Axonal voltage excursions are modeled at an individual node of Ranvier with the Hodgkin-Huxley model [85] using the values listed in Ochab-Marcinek et al [86]. We start with the basic equation for membrane potential (*V_m_*):
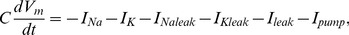
(1)where *C* is the nodal membrane capacitance. The total *I_Na_* for all Nav channels and the potassium current *I_K_* are given as:
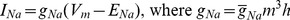
(2)

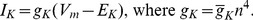
(3)

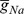
 and 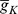 are the maximal conductances of the Nav and Kv channels, respectively; *E_Na_* and *E_K_* are the sodium and potassium reversal potentials, respectively; and *n^4^* gives the steady-state open probability of potassium channels. By the HH formulation, gating variables *m*, *h*, and *n* evolve according to:
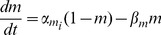
(4)

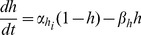
(5)

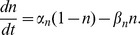
(6)The forward (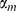 and 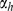) and backward rate functions (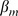 and 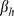) describe the first-order transitions between the deactivated and activated states of activation (*m*) and inactivation (*h*) processes in Eqs (4–5). They are determined by the membrane potential *V_m_*:
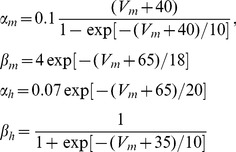
(7)The rate functions *α_n_* and *β_n_* of potassium gating variable *n* in Eq (6) also depend on *V_m_*:
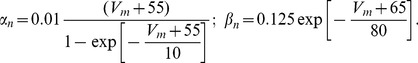
(8)Experimental findings for recombinant *I_Na_* from nodal type Nav1.6 channels [8] show that, with mechanical membrane damage (“injury”), transient *I_Na_* at a given *V_m_* irreversibly accelerates because the activation and steady state inactivation (availability) processes undergo irreversible hyperpolarizing (“left”)-shifts. Consistent with this, the steady-state product of activation and availability (*m^3^ h*(*V_m_*)*_t→∞_*), also called the window conductance, also left-shifts. This injury response we term “coupled left-shift” (CLS). This CLS can be easily modeled within the above HH model by shifting the voltage dependences of the activation (*m*) and inactivation (*h*) functions by *LS*, i.e replacing *V_m_* by (*V_m_*+*LS*) in Eqs (7). In other words whatever used to be observed at a given *V_m_* in any function of *m* and *h* is now observed at (*V_m_*−*LS*). This CLS is the basis of our model for axon damage. In patch clamp experiments the activation/inactivation time course data are explained with a single *LS* value for each degree of injury [8].

However, axon injury is spatially non-homogeneous [2] and is therefore likely to result in spatially inhomogeneous CLS. To account for this possibility we introduce fractions *f_i_* of Nav channels in damaged membrane, with left-shift intensity *LS_i_* (in millivolts). A Gaussian distribution of *LS_i_* seems a realistic model of axon injury, hence we explore various Gaussians. But discrete populations with a small number of *LS* values are more amenable to a thorough analysis, such as the bifurcation analysis done on a three population system exhibiting bursting dynamics (see Figure 5).

Specifically for a population of left-shifted Nav 1.6 channels {*f_i_*, *LS_i_*}_i = 1,N_, for each *f_i_* of intensity *LS_i_*, independent activation variables *m_i_* (i = 1,2,…N) and inactivation variables *h_i_* (i = 1,2,…N) are introduced. The total *I_Na_* for all Nav channels is now given as:
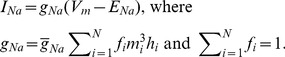
(9)


Three classes of Nav-CLS are considered here: (a) two *g_Na_* populations: one intact, one left-shifted by a specified fixed amount (as in [11]); (b) three populations: one intact, two with discrete *LS* values; and (c) assorted versions of *LS* of all or specified fractions of the total Nav population, but with *f_i_(LS)* following Gaussian distributions to mimic the expectation that nodal injury would produce “smeared” (as opposed to discrete) extents of “Nav-CLS”. At the node of Ranvier, *g_Na_* dominates over *g_K_*, but previously [11] we briefly discussed the effects of also applying a *LS* to the gating of the Kv channels.

The following features are also included in the model. Leakage currents [29], *I_Naleak_* and *I_Kleak_*, account for the background permeabilities needed to bring the basal system to a quiescent steady-state *V_m_* with an active pump present, along with the usual unspecific leakage current *I_leak_*. These leak currents take the form:
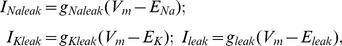
(9)where conductances *g_Naleak_*, *g_Kleak_* and *g_leak_* are constants. Parameter values are summarized in Table 1.

With an active Na/K pump, the model system has dynamic intracellular and extracellular Na^+^ and K^+^ concentrations, denoted by [*Na^+^*]_i_, [*Na^+^*]_o_, [*K^+^*]_i_, and [*K^+^*]_o_. The Na/K pump balances the accumulation and depletion of ions in the intra- and extracellular space by exchanging 3 internal Na^+^ for 2 external K^+^ ions. Thus [*K^+^*]_o_ and [*Na^+^*]_i_ determine the pump amplitude [29], [87]:
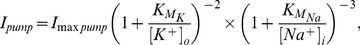
(10)where 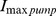 is the maximal current generated by the pump, 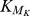 and 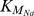 are Michaelis-Menten kinetic constants, and the *Na^+^* and *K^+^* currents flowing through the pump are *I_Napump_* = 3*I_pump_* and *I_Kpump_* = −2*I_pump_*. Ion fluxes across the node of Ranvier membrane cause the ion concentration changes:
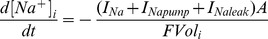


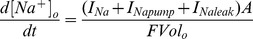
(11)

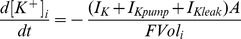


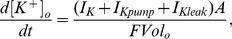
(12)where *F* is the Faraday constant, *A* is the surface area of the nodal membrane, and 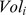 (or 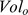) is the intracellular (or extracellular) volume of the node of Ranvier under study (we take 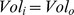 for simplicity). Ion concentration dynamics alter the reversal potentials *E_Ion_* in Eqs (2–3) by the Nernst equations:
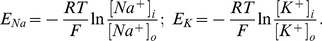
(13)Model equations are solved using a 4-th order Runge-Kutta scheme with 10^−3^ ms as time step in MATLAB and bifurcation diagrams were plotted using XPPAUT.
